# KDM2 Family Members are Regulated by HIF-1 in Hypoxia

**DOI:** 10.3390/cells6010008

**Published:** 2017-03-17

**Authors:** Michael Batie, Jimena Druker, Laura D’Ignazio, Sonia Rocha

**Affiliations:** Centre for Gene Regulation and Expression, School of Life Sciences, University of Dundee, Dow street, Dundee DD1 5EH, UK; m.t.batie@dundee.ac.uk (M.B.); j.druker@dundee.ac.uk (J.D.); l.dignazio@dundee.ac.uk (L.D.)

**Keywords:** hypoxia, HIF-1, KDM2, JmjC, drosophila, ChIP

## Abstract

Hypoxia is not only a developmental cue but also a stress and pathological stimulus in many human diseases. The response to hypoxia at the cellular level relies on the activity of the transcription factor family, hypoxia inducible factor (HIF). HIF-1 is responsible for the acute response and transactivates a variety of genes involved in cellular metabolism, cell death, and cell growth. Here, we show that hypoxia results in increased mRNA levels for human lysine (K)-specific demethylase 2 (KDM2) family members, KDM2A and KDM2B, and also for *Drosophila melanogaster* KDM2, a histone and protein demethylase. In human cells, KDM2 family member’s mRNA levels are regulated by HIF-1 but not HIF-2 in hypoxia. Interestingly, only KDM2A protein levels are significantly induced in a HIF-1-dependent manner, while KDM2B protein changes in a cell type-dependent manner. Importantly, we demonstrate that in human cells, KDM2A regulation by hypoxia and HIF-1 occurs at the level of promoter, with HIF-1 binding to the *KDM2A* promoter being required for RNA polymerase II recruitment. Taken together, these results demonstrate that KDM2 is a novel HIF target that can help coordinate the cellular response to hypoxia. In addition, these results might explain why KDM2 levels are often deregulated in human cancers.

## 1. Introduction

Hypoxia, or reduced oxygen availability, is an important developmental cue in multicellular organisms, but it is also involved in a number of human pathologies [1,2]. In response to hypoxia, cells orchestrate a tightly controlled and coordinated response, mostly dependent on transcriptional changes [3]. In the centre stage of such a response, stands the transcription factor family, hypoxia inducible factor (HIF).

HIF is a heterodimer consisting of a α and a β subunits, also known as aryl hydrocarbon nuclear translocator [4]. While HIF-1β protein levels remain largely unchanged in hypoxia, HIF-α levels are extremely sensitive to change in oxygen availability. Oxygen sensitivity is conferred to the HIF system, via the action of a class of dioxygenases called prolyl hydroxylases (PHDs). PHDs require molecular oxygen, 2-oxoglutarate, and iron as cofactors to perform proline hydroxylation of their targets [5]. Proline hydroxylation of two conserved residues within HIF-α creates a high-affinity binding site for a ubiquitin E3 ligase complex containing the tumour suppressor, von Hippel Lindau (VHL). VHL thus promotes the ubiquitination and proteasome-dependent degradation of HIF-α under normal oxygen conditions [6].

In addition to changes in HIF-α protein stability, an additional level of oxygen control is mediated by another dioxygenase called factor inhibiting HIF (FIH). FIH mediates asparagine hydroxylation in the transactivation domain of HIF-α, a modification that prevents efficient recruitment of coactivators such as p300/CBP to HIF-α, thus limiting the transcriptional output of HIF [7]. It has been shown that FIH is still functional at oxygen concentrations where PHDs are already inhibited [8]. Furthermore, genetic studies have shown that FIH regulates 40% of HIF-dependent genes [9]. However, FIH knockout mice develop normally, suggesting that FIH is not involved in controlling HIF-dependent transcription during developmental hypoxia [10].

Structural studies of the FIH enzyme revealed an interesting fold, identical to a domain called Jumonji C (JmjC) [11,12]. JmjC enzymes are protein demethylases, and a number of them possess histone demethylase activity [13]. As such, these enzymes have the potential of sensing and being controlled by oxygen levels in the cell. Changes in histone methylation have been described in hypoxia in a number of cellular systems [13,14]. One of the JmjC enzymes, lysine (K)-specific demethylase (KDM)4E, has been analysed in terms of its oxygen dependency, revealing a similar response to oxygen to that of PHDs [15]. Interestingly, some of the JmjC enzymes have been shown to be induced in hypoxia, and some in a HIF-dependent manner (reviewed in [13]). These include KDM3A, KDM4B and C, and KDM5C [16,17,18,19,20].

One interesting family of JmjC enzymes that is deregulated in human disease [13] is KDM2. KDM2A and KDM2B demethylate histone 3 lysine 36 when mono- and dimethylated [21,22]. The KDM2 family possesses additional functional domains to JmjC, including an Fbox domain, a PHD domain, a CXXC-zinc finger domain, and a leucine-rich repeat domain [13]. Apart from demethylating histone H3 tails, these proteins have also been shown to have non-histone targets such as RelA and β-catenin [23,24]. Furthermore, these enzymes have been shown to be deregulated in cancers such as breast and pancreatic [25,26,27].

Here, we demonstrate that KDM2A is hypoxia-inducible in a HIF-dependent manner. We find that HIF-1α regulates the *KDM2A* promoter and that HIF-1 is required for the recruitment of RNA polymerase II to the *KDM2A* promoter. Interestingly, although KDM2B mRNA is induced in a HIF-dependent manner in hypoxia, KDM2B protein level changes in hypoxia are dependent on the cell type analysed.

## 2. Material and Methods

Cells and cell culture: Human embryonic kidney cells HEK293, U2OS osteosarcoma, HeLa cervix carcinoma, and MCF-7 breast cancer cells were obtained from the European Collection of Cell Cultures and grown in Dulbecco’s Modified Eagle Medium (Lonza) supplemented with 10% foetal bovine serum (Gibco), 50 units/mL penicillin (Lonza), and 50 µg/mL streptomycin (Lonza) for no more than 30 passages at 37 °C and 5% CO_2_. Cells were routinely tested for contamination.

Hypoxia treatments: All hypoxia exposures were performed at 1% O_2_ in an in vivo 300 hypoxia workstation (Ruskin, Pencoe, UK). Cells were lysed for protein extracts, RNA extraction, immunofluorescence, and chromatin immunoprecipitation fixation in the workstation to avoid reoxygenation.

*Drosophila melanogaster* experiments: Fly culture and husbandry were performed according to standard protocols. All procedures were previously described in [28,29].

Antibodies: HIF-1α (610958, BD Biosciences San Jose, CA, USA, and sc-53546 Santa Cruz Biotechnology, Dallas, TX, USA), HIF-2α (sc-13596, Santa Cruz Biotechnology ), HIF-1β (#5537, Cell Signaling, Danvers, MA, USA), β-actin (3700, Cell Signaling), RNA polymerase II (sc-47701, Santa Cruz Biotechnology), KDM2A (A301-476A, Bethyl laboratories, Montgomery, TX, USA), and KDM2B (09-862, Millipore; GTX104868. Genetex, Zeeland, MI, USA).

Immunoblot: Cells were lysed in RIPA buffer, 50 mM Tris-HCl (pH 8), 150 mM NaCl, 1% (*v/v*) NP40, 0.5% (*v*/*v*) Na-deoxycholate, 0.1% (*v*/*v*) SDS, and 1 tablet/10 mL Complete, Mini, EDTA-free protease inhibitors (Roche). SDS-PAGE and immunoblots were carried out using standard protocols.

mRNA analysis: RNA was extracted using peqGOLD total RNA kit (Peqlab, Erlangen, Germany) or Direct-Zol RNA kit (Zymo Research, Irvine, CA, USA) according to the manufacturer’s instructions, and reverse transcribed using QuantiTect Reverse Transcription Kit (Qiagen, Hilden, Germany). For quantitative PCR, Brilliant II Sybr Green kit (Statagene/Agilent, Santa Clara, CA, USA), including specific MX3005P 96-well semi-skirted plates, were used to analyse samples on the MX3005P qPCR platform (Stratagene/Agilent). Actin was used as a normalising agent in all experiments. qPCR results were analysed by the ΔΔCt method. Control normoxia or hypoxia were used as calibrators in the different experiments, and set to 100.

The following primers were used for RT-PCR:

Actin

F: 5’-CTGGGAGTGGGTGGAGGC-3’, and

R: 5’-TCAACTGGTCTCAAGTCAGTG-3’.

KDM2A

F: 5’-CAGGAGGCCGGGCTCTCAGT-3’, and

R: 5’-CGGGTCTGGGACTCCTGGGG-3’.

HIF-1α

F: 5’-CATAAAGTCTGCAACATGGAAGGT-3’, and

R: 5’-ATTTGATGGGTGAGGAATGGGTT-3’.

HIF-1β

F: 5’-CAAGCCCCTTGAGAAGTCAG-3’; and

R: 5’-GAGGGGCTAGGCCACTATTC-3’.

CA9

F: 5’-CTTTGCCAGAGTTGACGAGG-3’; and

R: 5’-CAGCAACTGCTCATAGGCAC-3’.

HIF-2α

F: 5’-TTTGATGTGGAAACGGATGA-3’, and

R: 5’-GGAACCTGCTCTTGCTGTTC-3’

KDM2B and KDM2B isoform 1 validated primer set were obtained from Qiagen.

KDM2B_isoform2

F: 5’- AAGCAAGTCACCAAGGAAGG-3’; and

R: 5’- CCCAAACGGGTGGTTGAA-3’.

*Drosophila* primers:

drActin

F: 5’- GCGTTTTGTACAATTCGTCAGCAACC-3’,

R: 5’- GCACGCGAAACTGCAGCCAA-3’.

drKDM2

F: 5’-AGTGTTGAGGATAGCACGAAAG-3’,

R: 5’-CCCAGTAGCGTGTGAACATAG-3’.

siRNA: siRNA oligonucleotides were purchased from MWG and used in a final concentration of 27 nM. They were transfected using Interferin from Polyplus according to manufacturer’s instructions. The following oligonucleotides sequences were used for siRNA knockdown:

Control: 5’-AAC AGU CGC GUU UGC GAC UGG-3’,

HIF-1α: 5’-CUG AUG ACC AGC AAC UU-3’,

HIF-2α: 5’-CAG CAU CUU UGA CAG-3’.

HIF-1β: 5’-GGU CAG CAG UCU UCC AUG-3’.

KDM2B_1: 5’- CUA UGA GUA GGU ACA GAG A-3’

KDM2B_2: 5-CCA CUG CAA GUC UAG CAC A-3’

Chromatin immunoprecipitation: Following the different treatments, proteins and chromatin were cross-linked with 1% formaldehyde at room temperature for 10 min. Glycine was added to a final concentration of 0.125 M for 5 min to quench the reaction. Cells were harvested into 400 µL of lysis buffer (1% SDS, 10 mM EDTA, 50 mM Tris-HCl pH 8.1, 1 mM PMSF, 1 μg/mL leupeptin, 1 μg/mL aprotinin) and left on ice for 10 min. Samples were then sonicated at 4 °C eight times for 15 s with a 30 s gap between each sonication at 50% amplitude (Sonics Vibra-Cell # VCX130). Supernatants were recovered by centrifugation (12,000 rpm for 10 min at 4 °C) before 10% of each sample was stored as input. Remaining samples were split into 120 µL aliquots before being diluted 10-fold in dilution buffer (1% Triton X-100, 2 mM EDTA, 150 mM NaCl, 20 mM Tris-HCl pH 8.1). Diluted samples were precleared for 2 h at 4 °C by incubating with 2 µg of sheared salmon sperm DNA and 20 µL of protein G-Sepharose (50% slurry). Immunoprecipitations were performed overnight with 2 µg of specific antibody or IgG control, with the addition of Brij 35 detergent to a final concentration of 0.1%. Immune complexes were captured by incubation with 40 µL of protein G-Sepharose (50% slurry) and 2 µg salmon sperm DNA for 1 h at 4 °C. The immunoprecipitates were washed sequentially for 5 min each at 4 °C in Wash Buffer 1 (0.1% SDS, 1% Triton X-100, 2 mM EDTA, 20 mM Tris-HCl, pH 8.1, 150 mM NaCl), Wash Buffer 2 (0.1% SDS, 1% Triton X-100, 2 mM EDTA, 20 mM Tris-HCl, pH 8.1, 500 mM NaCl), and Wash Buffer 3 (0.25 M LiCl, 1% Nonidet P-40, 1% deoxycholate, 1 mM EDTA, 10 mM Tris-HCl, pH 8.1). Beads were washed twice with Tris-EDTA buffer and eluted with 120 µL of Elution Buffer (1% SDS, 0.1 M NaHCO_3_). Cross-links were reversed by incubation with 0.2 M NaCl at 65 °C overnight and Proteinase K (20 µg each), 40 mM Tris-HCl pH 6.5, and 10 mM EDTA for 1 h at 45 °C was used to remove protein. DNA was purified using a PCR-product purification kit according to manufacturer’s instructions (NBS Biologicals #NBS363). DNA (3 µL) was used for qPCR with the following primers for the putative hypoxia response element (HRE) sites at the *KDM2A* and *KDM2B* promoters:

KDM2A_HRE

F: 5’-GTCTGACGGGTTCAAAATGG-3’, and

R: 5’-CTACTCCCCAGTCGCAGAAG-3’.

KDM2B_HRE

F: 5’-CCTAGTAAAGGAGTCCACAG-3’, and

R: 5’-CCATACCTATAAGGACTGCC-3’.

Immunofluorescence: Cells were plated onto sterilised glass coverslips (VWR 19 mm) in 35 mm plates 24 h before transfection at a density of 1.2 × 10^5^ cells in a total volume of 2 mL media. Forty-eight hours following transfection, cells were fixed to coverslips in 100% methanol for 5 mins at −20 °C. Cells were then washed with PBS and blocked with 1% donkey serum in PBS/0.05% Tween for 30 min at room temperature. Cells were placed in a humidified chamber, incubated with primary antibody for 1 h, washed three times (5 min each) in PBS, incubated for 1 h with secondary antibody, and washed three times (5 min each) in PBS. Nuclear staining was performed by incubation with 33.3 μM Hoechst (Life Technologies) for 2 min. Coverslips were then washed in dH_2_O and mounted onto VWR SuperFrost slides using mounting medium (H-1000; Vector labs, Peterborough, UK) and sealed with nail varnish. Cell images were acquired using a DeltaVision microscope. Images were deconvolved and analysed using OMERO client software (5.2.7, Open Microscopy Environment, Dundee, UK) [30].

Plasmids and cloning: HIF-1α expression plasmid was obtained from Origene.

KDM2A HRE sites were cloned using *Kpn*I and *Mlu*I restriction enzymes in the pGL3 promoter luciferase construct (Promega) using the following oligonucleotides

WT KDM2A (−186 GCGTG, −143 GCGTG)

Forward: 

5’-CGCATCCGCGCGTGCGCGCGCGGAGTGACGCGTGCGCGTTCA-3’

Reverse: 

5’-CGCGTGAACGCGCACGCGTCACTCCGCGCGCGCACGCGCGGATGCGGTAC-3’

KDM2A −186 mut (−186 GATAA, −143 GCGTG)

Forward:

5’-CGCATCCGCGATAACGCGCGCGGAGTGACGCGTGCGCGTTCA-3’

Reverse:

5’-CGCGTGAACGCGCACGCGTCACTCCGCGCGCGTTATCGCGGATGCGGTAC-3’

KDM2A double mut (−186 GATAA, −143 GATAA)

Forward:

5’-CGCATCCGCGATAACGCGCGCGGAGTGACGATAACGCGTTCA-3’

Reverse:

5’-CGCGTGAACGCGTTATCGTCACTCCGCGCGCGTTATCGCGGATGCGGTAC-3’

Luciferase reporter assay: Cells (2 × 10^5^) were seeded in six-well plates and transfected with 1 µg DNA. At 48 h post-transfection, cells were lysed in 400 µL passive lysis buffer (Promega). Luciferase assays were performed according to the manufacturer's instructions (Luciferase Assay System, Promega). Results were normalised for protein concentration with all experiments being performed a minimum of three times before calculating means and standard error of the means.

Statistical analysis: Student t-tests were performed for all the data comparing control to treatment conditions, with values calculated: p values, with * *p* < 0.05, ** *p* < 0.01, and *** *p* < 0.001.

## 3. Results

### 3.1. Hypoxia Induces Changes in KDM2A and KDM2B mRNA Levels

**Figure 1:**
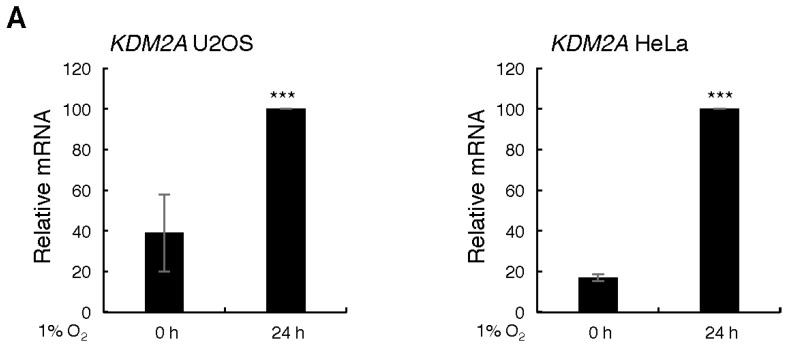

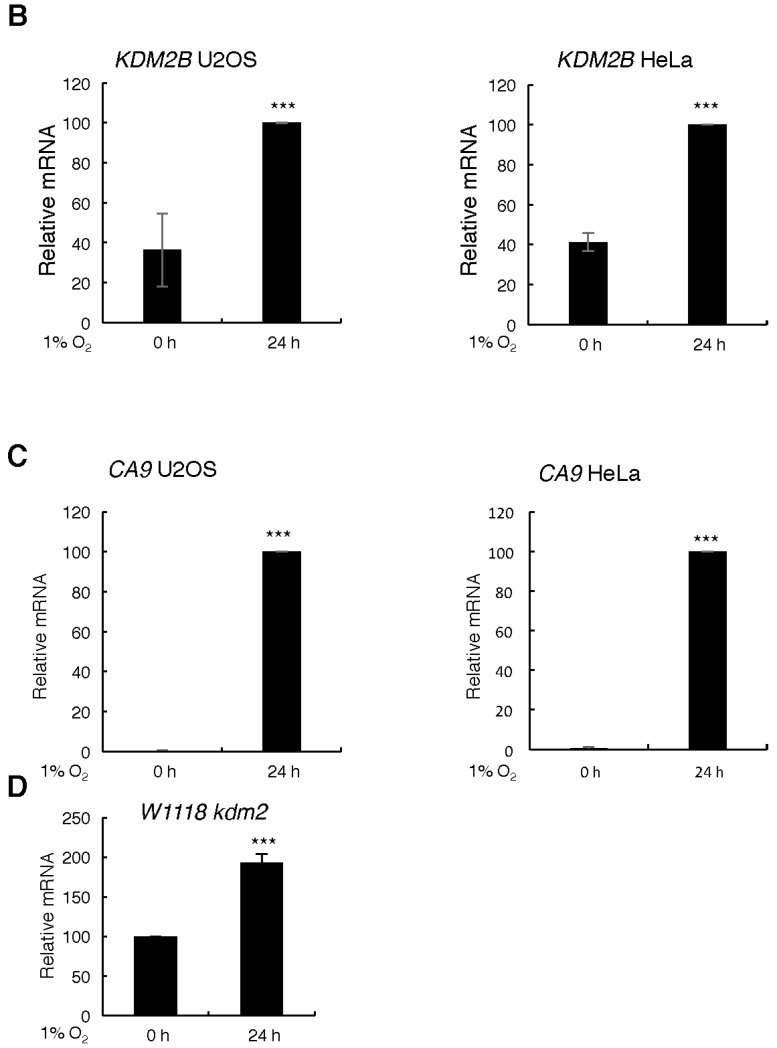
KDM2 mRNA is induced in hypoxia in human and in *Drosophila melanogaster*. U2OS and HeLa cells were exposed to 1% O_2_ for 24 h prior to RNA extraction. Following cDNA synthesis, qPCR analysis was performed for the levels of (**A**) KDM2A, (**B**) KDM2B, and (**C**) CA9 mRNA. Graphs depict mean and standard deviation from a minimum of three independent experiments performed in duplicate. (**D**) Adult animals were exposed to 5% O_2_ for 24 h prior to total RNA extraction. Levels of KDM2 were as in (**A**). Graph depicts mean and standard deviation of two independent experiments consisting of 70 animals per condition. Student t-test was performed and *p* values calculated. *** *p* < 0.001.

In order to determine if KDM2 family members are transcriptionally regulated in hypoxia, U2OS and HeLa cells were exposed to 1% O_2_ for 24 h prior to mRNA extraction and qPCR analysis. Levels of KDM2A and KDM2B were analysed (Figure 1A,B) and CA9, a known HIF-dependent target, was used as a control (Figure 1C). Both KDM2A and KDM2B mRNA were induced by hypoxia (Figure 1A,B). As expected, CA9 mRNA was robustly induced in both cell lines following hypoxia (Figure 1C). In addition, we also investigated if hypoxia was able to induce KDM2 in the model organism *Drosophila melanogaster*, where only one gene is present. Here, whole flies were exposed to 5% oxygen for 24 h prior to mRNA extraction and gene expression analysis. As it can be seen in Figure 1D, hypoxia induces the levels of KDM2 mRNA in this organism as well.

### 3.2. Hypoxia Induces KDM2A Protein but KDM2B Changes are Cell Type-Dependent

**Figure 2:**
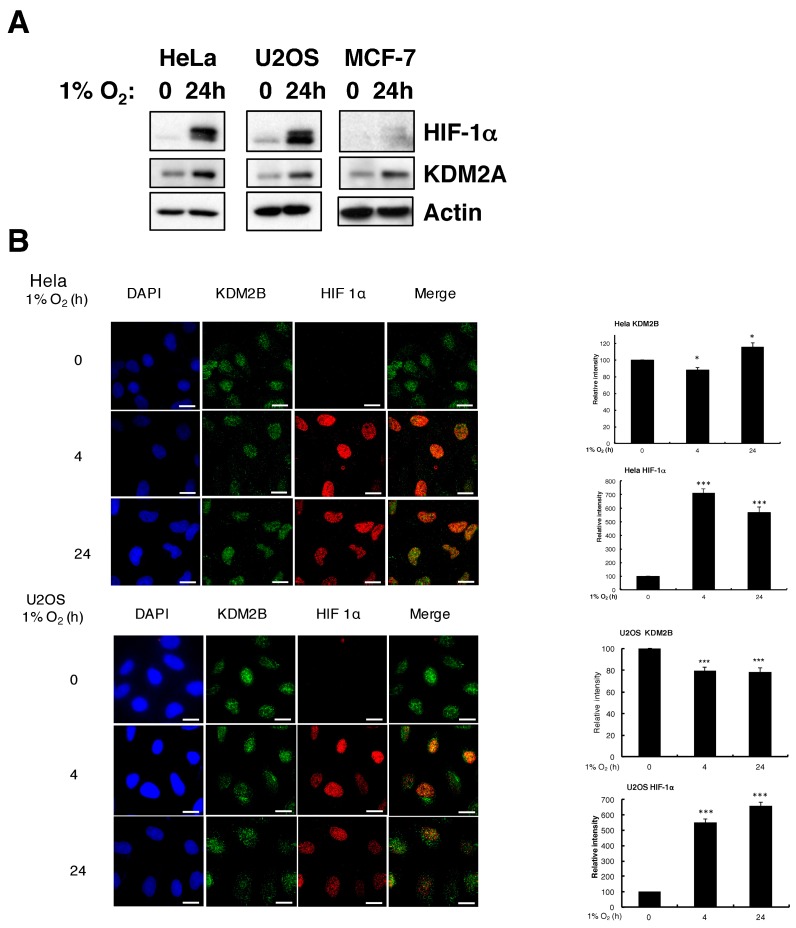

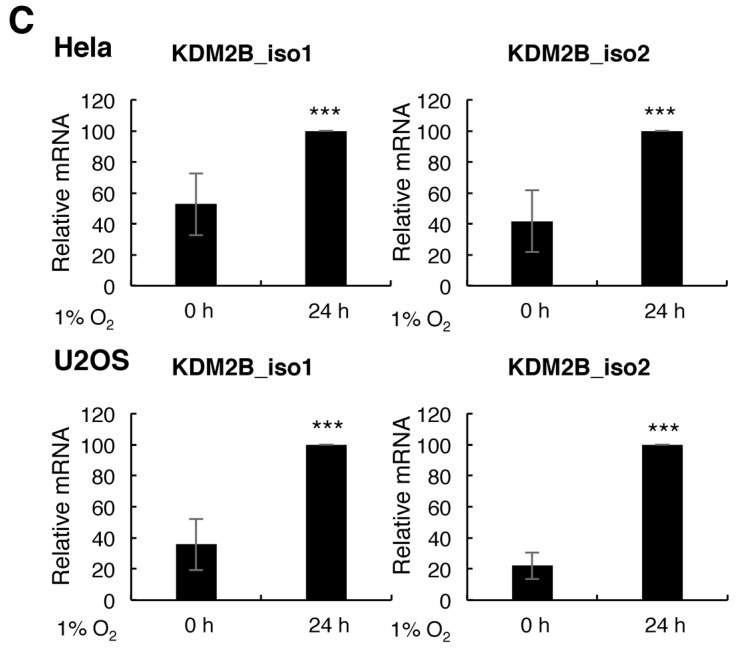
Hypoxia induces KDM2A protein but has little effect on KDM2B protein levels. (**A**) U2OS and HeLa cells were exposed to 1% O_2_ for the depicted times prior to cell lysis. Western blot was performed for the levels of the indicated proteins. Nonspecific band is labelled ns; (**B**) HeLa and U2OS were grown on coverslips and exposed to 1% O_2_ for the times depicted prior to fixation with methanol. Cells were stained with anti-KDM2B, HIF-1α antibodies, and DAPI to mark chromatin. Scale bar represents 10 µm. Images were acquired using a Deltavision microscope, and deconvolved and analysed using Omero software. Pixel intensities were quantified in Omero using the region of interest (ROI) tool. Graph depicts mean and standard deviation (SD) of a minimum of 100 cells per condition. Student *t*-test was performed and *p* value calculated. * *p* < 0.05, *** *p* < 0.001.

Next, we assessed if hypoxia could induce KDM2A and KDM2B protein levels. Western blot analysis revealed that, indeed, hypoxia induces KDM2A protein (Figure 2A) in the cell lines analysed. While numerous attempts were made to investigate KDM2B protein levels by Western blot, none of the antibodies tested were specific for KDM2B when an siRNA depletion control was included (Figure S1A), despite all the antibodies presenting a band of the expected size (Figure S1B–D). However, we could determine that one particular antibody was specific when used in immunofluorescence, accessed by siRNA depletion, overexpression of KDM2B, and specific controls for antibody staining (Figure S2A–C). 

Using immunofluorescence (IF), we find that hypoxia exposure results in reduced expression of KDM2B protein in U2OS cells and in a small statistical upregulation in HeLa cells following 24 h of hypoxia, assessed by analysing the intensity of antibody staining across several cells (Figure 2B). 

Given that KDM2B possesses two different isoforms [31], we investigated the possibility that our mRNA analysis was only measuring one specific isoform, not recognised by the antibodies we have tested. As such, we designed specific PCR primers directed at each of the KDM2B isoforms and repeated our qPCR analysis in cells exposed or not to hypoxia. This analysis revealed that hypoxia induces the expression of both KDM2B transcripts significantly in both HeLa and U2OS (Figure 2C). This confirmed our initial mRNA analysis for total KDM2B mRNA, and suggests that hypoxia induces changes in KDM2B protein at the post-transcriptional level.

### 3.3. Changes to the Levels of KDM2 Family Members in Hypoxia are HIF-1 Dependent

**Figure 3:**
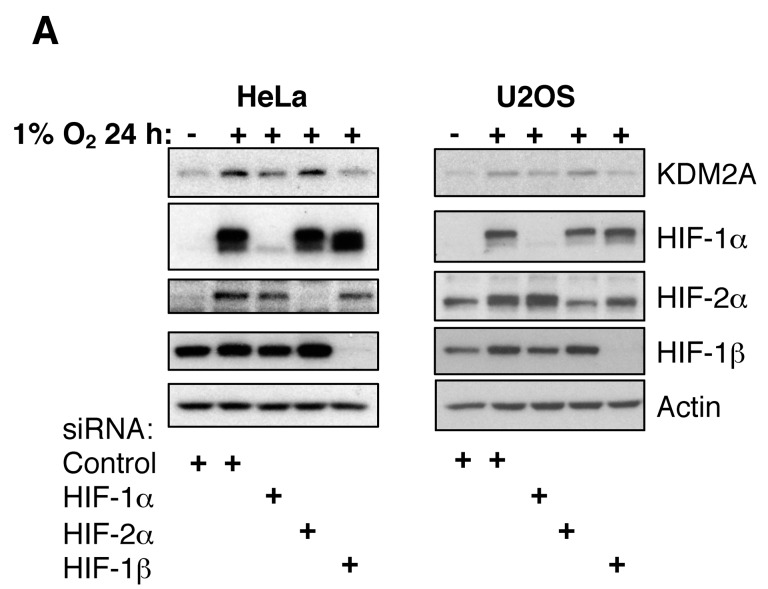

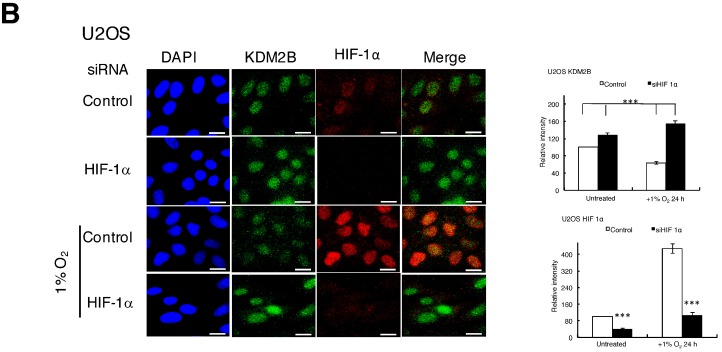
Hypoxia-induced KDM2A protein requires HIF-α/HIF-1β heterodimer. (**A**) U2OS and HeLa cells were transfected with the indicated siRNA oligonucleotides for 48 h prior to lysis. Where indicated, cells were also exposed to 1% O_2_, for 24 h, prior to lysis. Cell lysates were analysed by Western blot using the indicated antibodies. (**B**) U2OS cells were grown on cover slips and transfected with the indicated siRNA oligonucleotides prior to methanol fixation. Where indicated, cells were also exposed to 1% O_2_, for the periods of time depicted, prior to fixation. Cells were stained with anti-KDM2B, HIF-1α antibodies, and DAPI to mark chromatin. Scale bar represents 10 µm. Images were acquired using a Deltavision microscope, and deconvolved and analysed using Omero software. Pixel intensities were quantified in Omero using the ROI tool. Graph depicts mean and standard deviation (SD) of a minimum of 100 cells per condition. Student t-test was performed and *p* values calculated. *** *p* < 0.001.

Given that hypoxia induces changes to KDM2 proteins, we next determined if these were HIF dependent. First, we analysed KDM2A protein levels in response to hypoxia in the presence or absence of HIF subunits. Western blot analysis revealed that KDM2A protein levels were induced in hypoxia in a HIF-1α-, HIF-1β-dependent manner, while HIF-2α did not contribute to these effects (Figure 3A). Interestingly, KDM2B protein levels are also HIF-1α dependent, as HIF-1α depletion resulted in higher KDM2B protein levels both in normoxia and especially in hypoxia in U2OS cells (Figure 3B). This data was also observed when additional siRNA oligonucleotides were used in HeLa cells (Figure S3A). 

**Figure 4:**
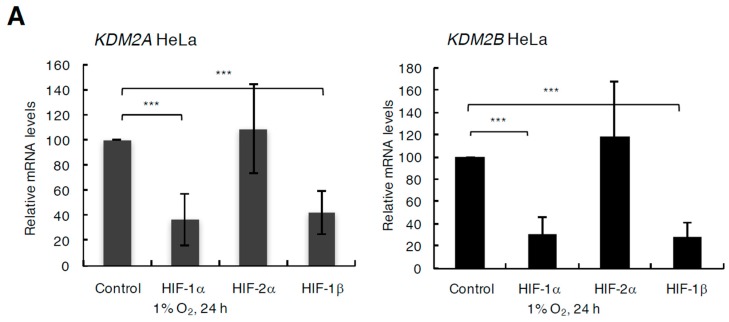

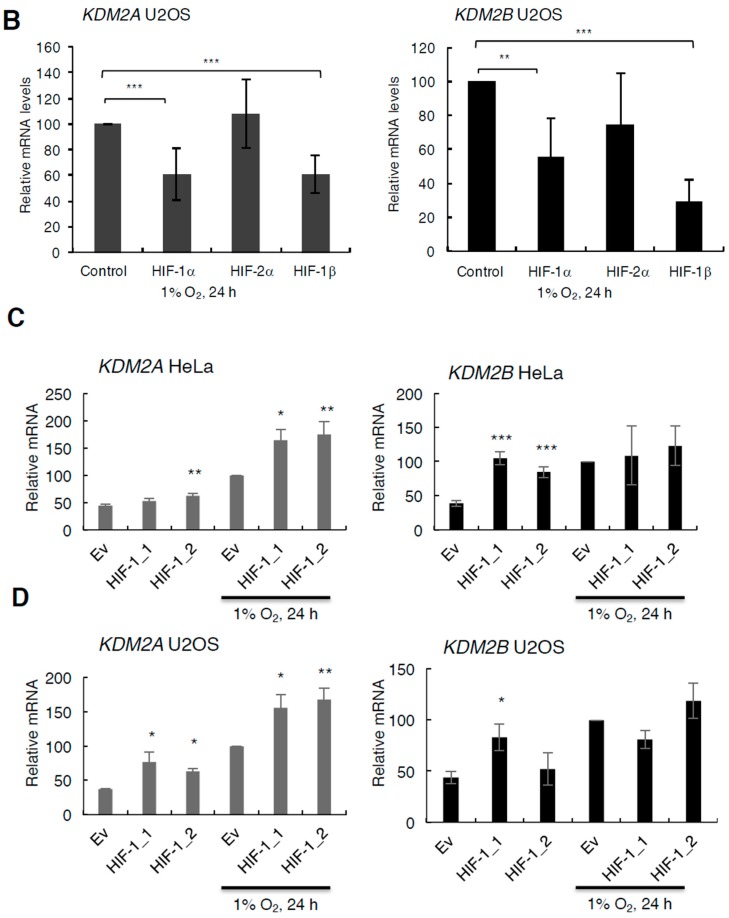
Hypoxia-induced KDM2A and KDM2B mRNA in a HIF-α/HIF-1β-dependent manner. (**A**) HeLa and (**B**) U2OS cells were transfected with the indicated siRNA oligonucleotides for 48 h prior to RNA extraction. In addition, cells were exposed to 1% O_2_ for the last 24 h. Following cDNA synthesis, qPCR analysis was performed for the levels of KDM2A and KDM2B mRNA. Graphs depict mean and standard deviation from a minimum of three independent experiments performed in duplicate. Student t-test was performed and *p* values calculated. * *p* < 0.05, ** *p* < 0.01, *** *p* < 0.001. (**C**) HeLa and (**D**) U2OS cells were transfected with empty vector (EV), or HIF-1α (1 or 2 µg) for 48 h prior to RNA extraction. In addition, cells were exposed to 1% O_2_ for the last 24 h. Following cDNA synthesis, qPCR analysis was performed for the levels of KDM2A and KDM2B mRNA. Graphs depict mean and standard error of mean from a minimum of three independent experiments performed in duplicate. Student t-test was performed and *p* values calculated. * *p* < 0.05, ** *p* < 0.01, *** *p* < 0.001.

As HIF is a transcription factor, we next analysed the levels of KDM2A and KDM2B mRNAs in the presence or absence of HIF subunits (Figure 4). Both KDM2A and KDM2B mRNA levels in HeLa and U2OS cells were dependent on the presence of HIF-1α and HIF-1β in hypoxia (Figure 4A,B, Figure S4A). These results suggest that KDM2 family is transcriptionally regulated by HIF-1, which results in changes in protein levels in hypoxia. Similarly, overexpression of HIF-1α could induce KDM2A mRNA levels in both cells lines in normoxia and hypoxia (Figure 4C, Figure S4B). Interestingly, HIF-1α overexpression also resulted in increased levels of KDM2B, but this was only observed under normoxic conditions (Figure 4C, Figure S4B).

### 3.4. KDM2A Promoter is Regulated by HIF-1

**Figure 5:**
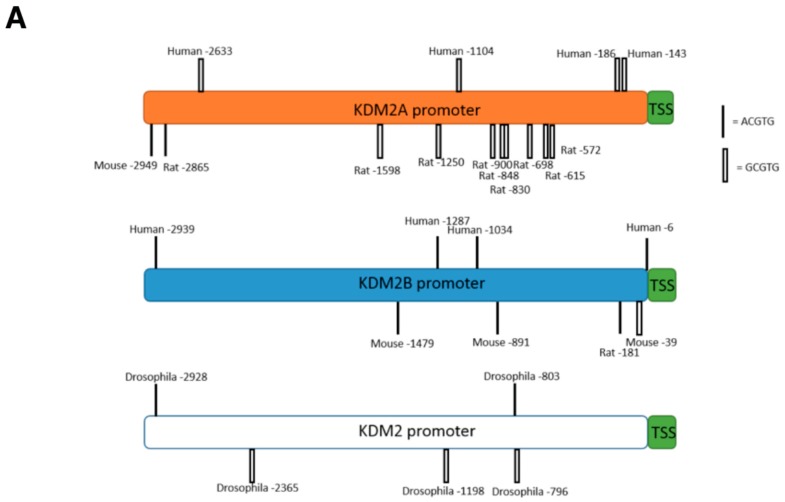

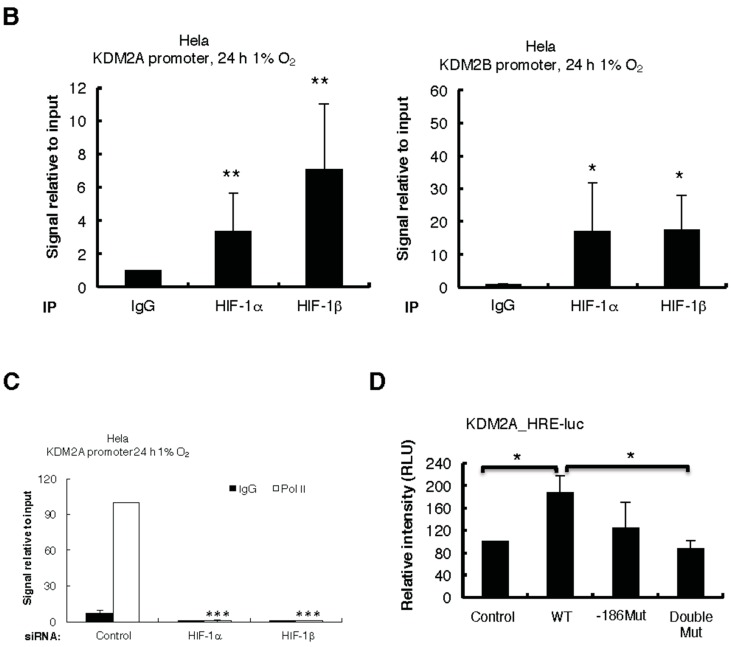
HIF-1α/HIF-1β heterodimer is present at the *KDM2A* promoter and is required for RNA polymerase II recruitment. (**A**) Schematic diagram of the *KDM2A* and *KDM2B* genes and annotation of putative hypoxia responsive elements (HREs) in different species; (**B**) HeLa cells were exposed to 1% O_2_ for 24 h prior to crosslinking and lysis. Chromatin immunoprecipitations (ChIPs) were performed for the levels of HIF-1α and HIF-1β present at the *KDM2A* and *KDM2B* promoters. IgG was used as the antibody control; (**C**) HeLa cells were transfected with the indicated siRNA oligonucleotides for 72 h prior to cross-linking and lysis. In addition, cells were exposed to 1% O_2_ for the last 24 h. ChIP was performed for the level of RNA polymerase II present at the *KDM2A* promoter, with IgG used as the antibody control. Graphs depict mean and standard deviation from a minimum of three independent experiments performed in duplicate. Student *t*-test was performed and *p* values calculated. * *p* < 0.05, ** *p* < 0.01, *** *p* < 0.001; (**D**) HEK293 cells were transfected with 500 ng PGL3 (control) vector or KDM2A HRE–luciferase constructs as indicted for 48 h prior to lysis and luciferase activity measured. Graph depicts mean and standard error of mean of a minimum of three independent experiments. Student *t*-test was performed and *p* values calculated. * *p* < 0.05, ** *p* < 0.01, *** *p* < 0.001.

Given our results, we next determined if HIF-mediated regulation of KDM2 was occurring at the promoter level. Bioinformatic analysis of the *KDM2A* and *KDM2B* promoters revealed putative hypoxia response elements (HREs) in both promoters, some quite close to the transcriptional start site (Figure 5A, Figure S5). We thus designed primer sets for the putative HRE sites present in the *KDM2A* and *KDM2B* promoters nearer the transcription start and performed chromatin immunoprecipitation (ChIP) analysis in hypoxia, for the levels of HIF-1α and HIF-1β present at these sites using qPCR (Figure 5A). We could detect significant levels of both HIF-1α and HIF-1β present at the *KDM2A* promoter when cells were exposed to hypoxia (Figure 5A). Interestingly, HIF-1α and HIF-1β were also present at the *KDM2B* promoter, under the same conditions.

As we had observed HIF-1-dependent increases in mRNA and protein of *KDM2A* in hypoxia, we next investigated the functional significance of HIF-1 presence at the *KDM2A* promoter. To this end, HIF-1 subunits were depleted by siRNA from cells and levels of RNA polymerase II present at the *KDM2A* promoter were analysed by ChIP-qPCR. This analysis was performed following 24 h of hypoxia, where we had observed the highest mRNA induction, in a HIF-dependent manner. Levels of RNA polymerase II were robustly detected in control cells exposed to hypoxia, when the *KDM2A* promoter was analysed (Figure 5B). However, when either HIF-1α or HIF-1β were depleted by siRNA from cells, levels of RNA polymerase II present at the *KDM2A* promoter dropped significantly and to almost background levels (Figure 5B). These results demonstrate the importance of HIF-1 subunits in the regulation of the *KDM2A* promoter, indicating that HIF-1 is required for transcriptional activation of this gene. To further determine the functionality of the HRE sites present at the *KDM2A* promoter, we created luciferase constructs expressing the two putative HRE sites in tandem, as well as mutant versions of the HRE sites. Analysis of luciferase activity in cells revealed that indeed these HREs confirm promoter activity (Figure 5D). Furthermore, this analysis revealed that both HREs are required for full luciferase activity, since, when mutated, luciferase output reverts to background level, while mutation of the −186 HRE site gives an intermediate activity level (Figure 5D).

Taken together, these results indicate that KDM2 family members are novel HIF-1 targets in response to hypoxia, and might explain why KDM2 levels are often deregulated in human cancers.

## 4. Discussion

In this report, we have identified that the KDM2 family of JmjC demethylases is responsive to hypoxia, and that this is conserved in the model organism *Drosophila melanogaster*. Furthermore, we show that both KDM2A and KDM2B protein levels change in response to hypoxia in a HIF-1 dependent manner. KDM2A mRNA and protein are increased in response to hypoxia by a mechanism dependent on the HIF-1 heterodimer. However, while KDM2B mRNA is also increased in response to hypoxia, KDM2B protein is reduced in a HIF-1-dependent manner by a post-transcriptional mechanism in U2OS cells, while KDM2B protein levels are slightly higher in HeLa cells exposed to hypoxia. 

HIF-1 has been shown to regulate certain JmjC enzymes previously. As such, KDM3A, KDM4B, KDM4C, KDM5C, and KDM6B have all been shown to be HIF targets (reviewed in [13]). However, despite some reports indicating that KDM2A and KDM2B mRNA levels increase in response to hypoxia [14], there was no information as to whether these where HIF-dependent genes.

KDM2B has been previously shown to be nuclear factor (NF)-κB dependent, and be negatively regulated by TRAIL treatment in several cancer cell lines [32]. While we have previously shown that hypoxia activated NF-κB to modulate its targets [33], we have not investigated if hypoxia-induced KDM2B mRNA is NF-κB dependent. We did find that depletion of HIF-1 significantly decreased KDM2B mRNA in hypoxia. Moreover, recently, we demonstrated that HIF-1α acts as a repressor of NF-κB in cells, controlling its activity both in normoxia and hypoxia [29], thus suggesting that under these conditions, NF-κB does not control *KDM2B* expression. Intriguingly, hypoxia exposure results in reduced protein levels of KDM2B in U2OS, in a HIF-1-dependent manner, suggesting that HIF-1-mediated effects on KDM2B protein are not direct and are post-transcriptional. How KDM2B protein is regulated in U2OS cells is currently unknown and would thus require additional work.

Our data clearly show that KDM2A is a novel HIF-1α-dependent target. Transcript and protein levels increase in hypoxia in a HIF-1α-dependent manner in both cell lines tested. In addition, we could demonstrate that HIF-1α and HIF-1β are present at the *KDM2A* promoter and that this is required for recruitment of RNA polymerase II to this promoter. A study analysing RNA polymerase II behaviour in hypoxia has revealed that some promoters already possess RNA polymerase loaded but it was non-processive [34]. Hypoxia was shown to release RNA polymerase from these promoters and thus induce transcription of these targets [34]. However, the authors did mention exceptions such as ADM, for example, where hypoxia induced RNA polymerase II loading [34]. Furthermore, given that the resolution of the ChIP-qPCR is around 400 bp, our results could also be consistent with a HIF-dependent RNA polymerase II release as proposed by the abovementioned study.

KDM2A has been reported to act as an oncogene in different types of cancer. As such, it has been shown to promote tumourigenesis in lung [35] and gastric [36] cancers, and more recently an oncogenic isoform of KDM2A has been identified in breast cancer [26]. However, KDM2A has also been shown to repress rRNA transcription, a process required for cell proliferation [37,38]; also, when phosphorylated by ataxia-telangiectasia mutated (ATM), KDM2A helps in DNA damage repair [39]. Interestingly, HIF-1α also has dual roles in cancer, being important for tumourigenesis in cancer such as colon and breast [2,40], but acting more as a tumour suppressor in cancers such as renal clear cell cancer [41].

Given that KDM2 enzymes require oxygen for their histone demethylase function, it would be tempting to speculate that, despite the increase in expression, their demethylase activity would still be low. However, their other protein domains would still be functional, and as such their role as ubiquitin ligases might be increased in hypoxia. However, currently very few targets are known for these proteins and, as such, further work directed at these questions is therefore need. It would thus be interesting to analyse which proteins KDM2 family members interact with and modify, and what the functional consequences are for the cell, when KDM2 levels increase or decrease. Given that the KDM2 family targets important histone methylation marks such histone H3 lysine 4 trimethylation (H3K4me3) and H3K36me/me2 [13], regulation of KDM2 abundance will impinge into the levels of these histone marks. This suggests that prolonged hypoxia could therefore change chromatin structure via the mechanism described here, where HIF-1 induces KDM2 protein changes. Analysis of histone marks in cells exposed to different periods of hypoxia would allow to determine how the action of KDM2 proteins is controlled under such stress conditions.

## Supplementary Materials

The following are available online at www.mdpi.com/ 2073-4409/6/1/8/s1. Figure S1. siRNA and antibody validation for KDM2B. Figure S2. siRNA and antibody validation for KDM2B using immunofluorescence. Figure S3. Additional siRNA oligonucleotide for HIF subunit knockdown. Figure S4. HIF subunit RNA level controls. Figure S5. HRE annotation in the promoters of KDM2 family in different species.
