# Supplementary material for: KDM2 Family Members are Regulated by HIF-1 in Hypoxia
Source: Cells. 2017 Mar 17;6(1):8. doi: 10.3390/cells6010008 (PMC5371873; doi:10.3390/cells6010008)
Supplement: Supplementary file 1 [file cells-06-00008-s001.pdf]

Supplementary material for :

Regulation of KDM2 family gene expression by hypoxia.

Michael Batie§, Jimena Druker§, Laura D'Ignazio, and Sonia Rocha*

Centre for Gene Regulation and Expression,

School of Life Sciences, University of Dundee, Dow street,

Dundee DD1 5EH United Kingdom.

§these authors contributed equally to the study

*correspondence to: Sonia Rocha

Tel:+441382385792

Fax:+441382386375

e-mail: s.rocha@dundee.ac.uk

Supplementary Figure Legends

Figure S1

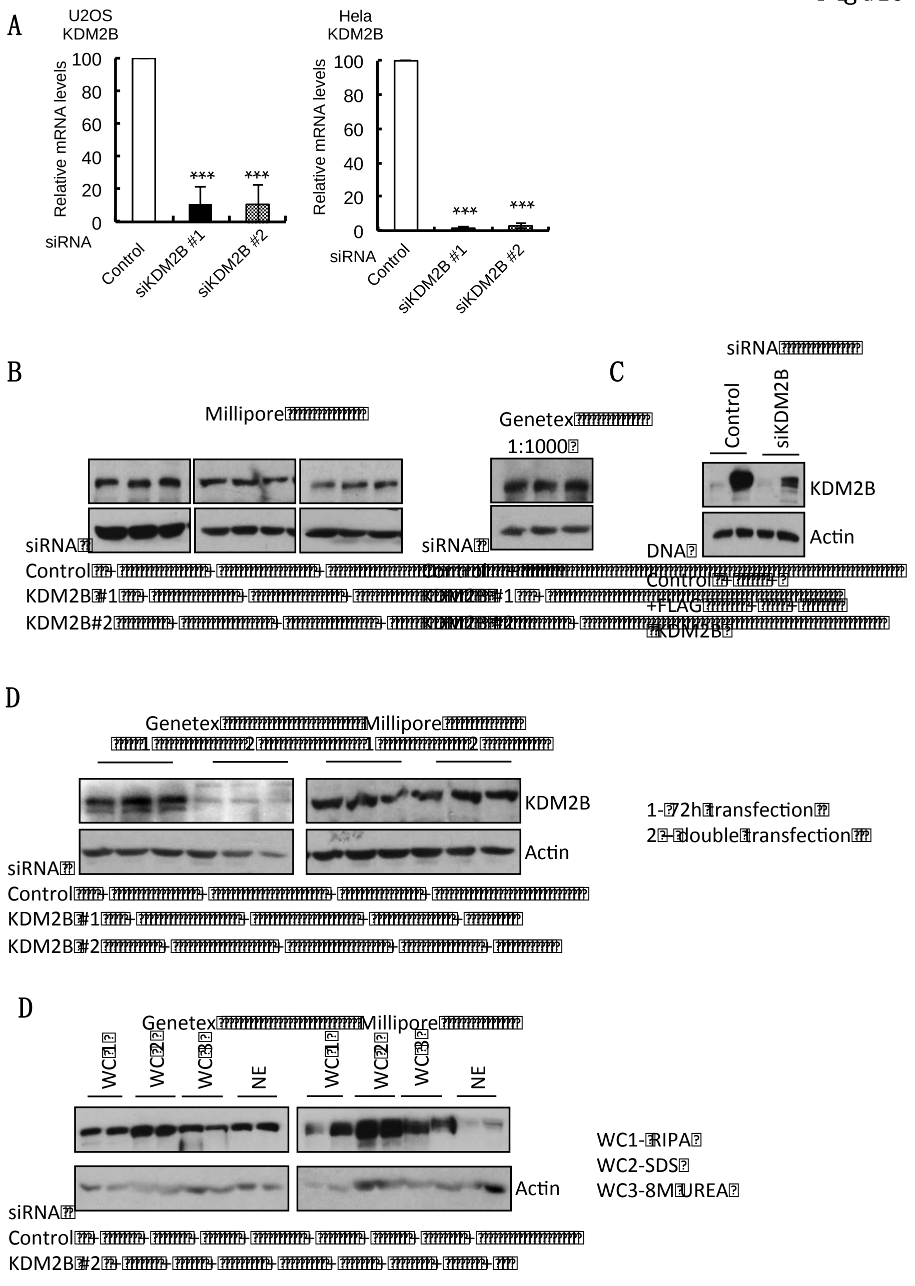

Sup Fig S1 siRNA and antibody validation for KDM2B. A. U2OS and HeLa cells were transfected with the indicated siRNA oligonucleotides for 48 hours prior to RNA extraction. Following cDNA synthesis, qPCR analysis was performed for the levels of KDM2B mRNA. Graphs depict mean and standard deviation from a minimum of three independent experiments performed in duplicate. Student t-test was performed and p values calculated. * $p< 0.05$, ** $p < 0.01$, *** $p<0.001$. B. HeLa cells

were transfected with the indicated siRNA oligonucleotides for 48 hours prior to lysis. Western blot analysis was performed with the depicted antibodies. C. HeLa cells were transfected with 1 μg of control or KDM2B expression plasmids for 24 hours prior to transfection with siRNA oligonucleotides as in A. Whole cell lysates were analysed by western blot for the levels of KDM2B using the Millipore antibody. D. HeLa cells were transfected with the indicated siRNA oligonucleotides for 72 hours or double siRNA transfection prior to lysis. Western blot analysis was performed with the depicted antibodies. E. HeLa cells were treated as in A, but different lysis buffers were used or nuclear extraction performed (NE). Lysates were analysed for the levels of KDM2B using the Millipore antibody.

Figure S2

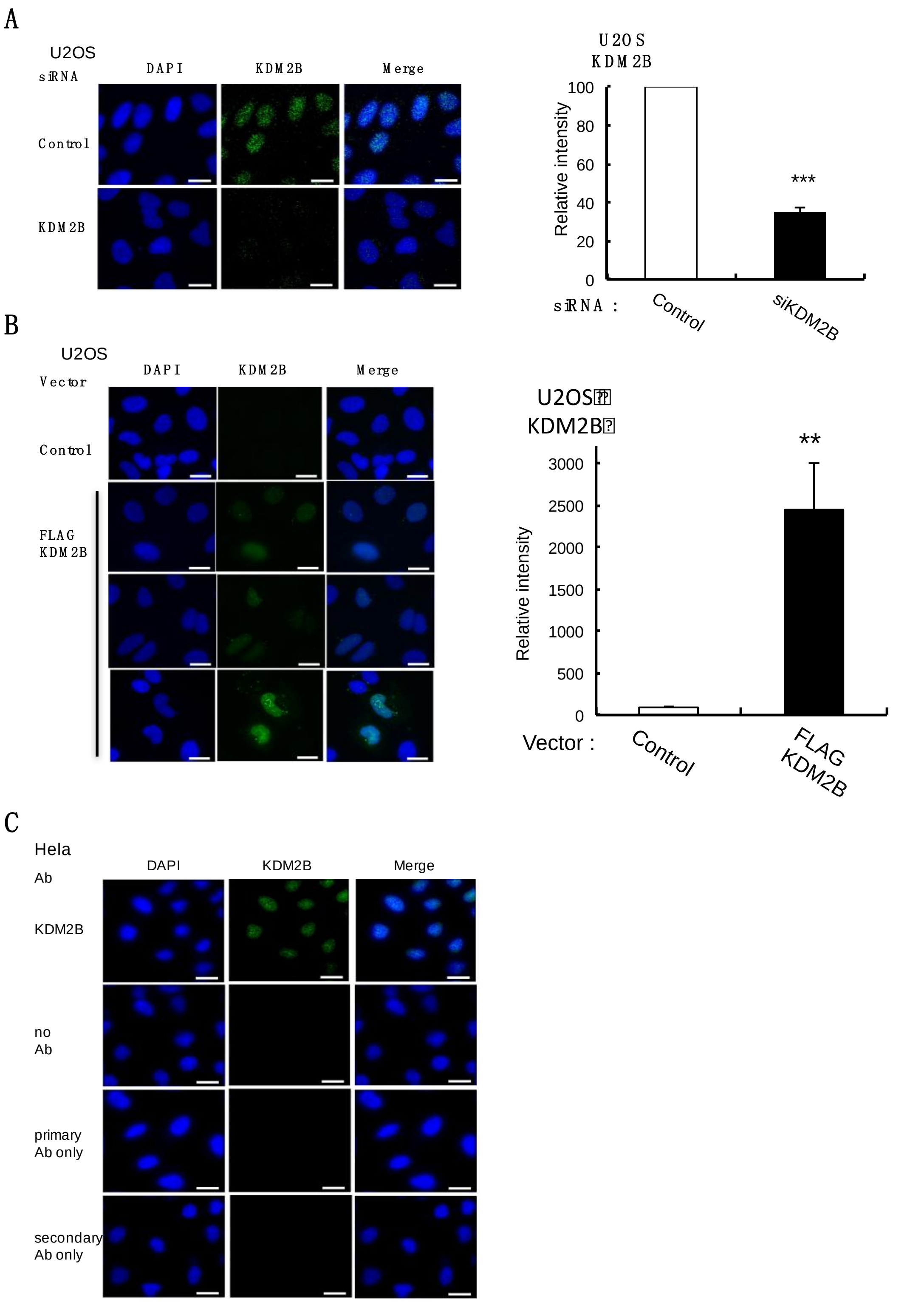

Sup Fig S2 siRNA and antibody validation for KDM2B using immunofluorescence.

A. U2OS cells were grown on cover slips and transfected with the indicated siRNA oligonucleotides prior to methanol fixation. Cells were stained with anti-KDM2B and

DAPI to mark chromatin. Scale bar represents 20μm. Images were acquired using a Deltavision microscope, deconvolved and analysed using Omero software. Pixel intensities were quantified in Omero using the ROI tool. Graph depicts mean and standard deviation (SD) of a minimum of 100 cells per condition. Student t-test was performed and p values calculated. * $p< 0.05$, ** $p < 0.01$, *** $p<0.001$. B. U2OS cells were grown on cover slips and transfected with the indicated expression vectors for 48 hours prior to methanol fixation. Cells were stained with anti-KDM2B and DAPI to mark chromatin. Scale bar represents 20μm. Images were processed and analyzed as in A. C. HeLa cells were grown on cover slips for 24 hours prior to methanol fixation. Cells were stained with anti-KDM2B and secondary antibody as standard, or just anti-KDM2B and no secondary antibody, or with secondary antibody only. DAPI was used to stain DNA. Scale bar represents 20μm. Images were processed as in A.

Figure S3

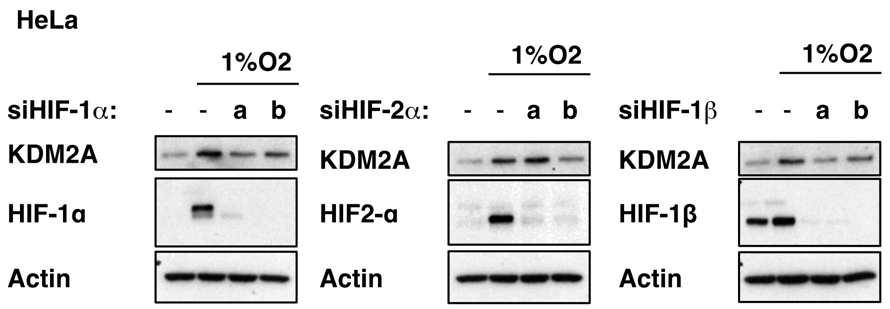

Sup Fig S3 Additional siRNA controls for HIF subunits. HeLa cells were transfected with the indicated siRNA oligonucleotides for 48 hours prior to lysis. In addition, cells were exposed to 1% O2 for the last 24 hours. Whole cell extracts were analysed by Western blot using the indicated antibodies.

Figure S4

A

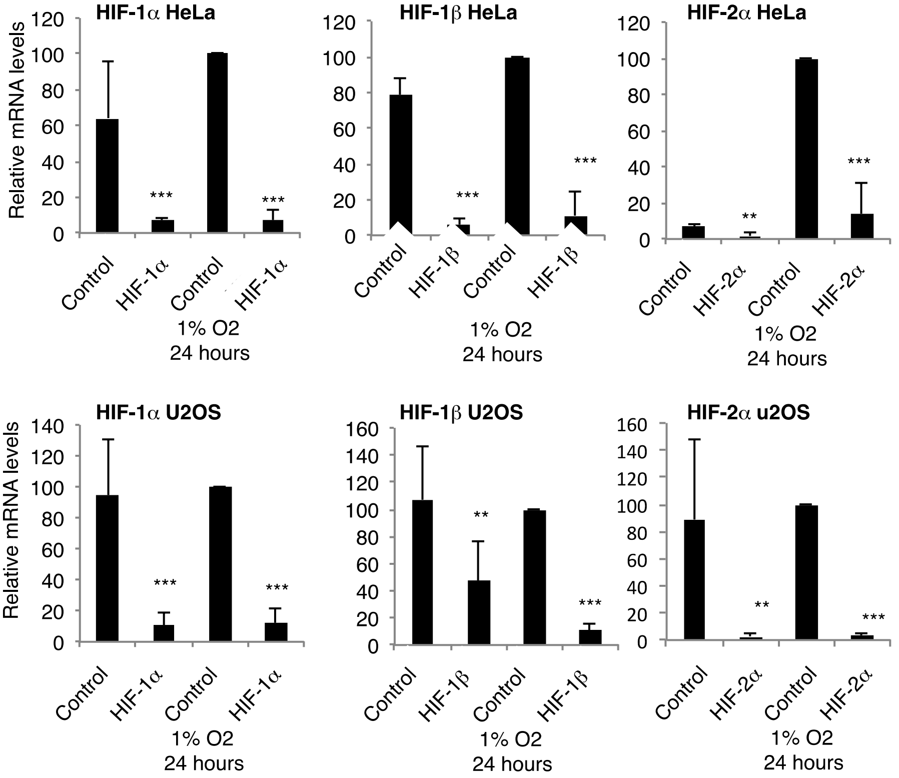

B

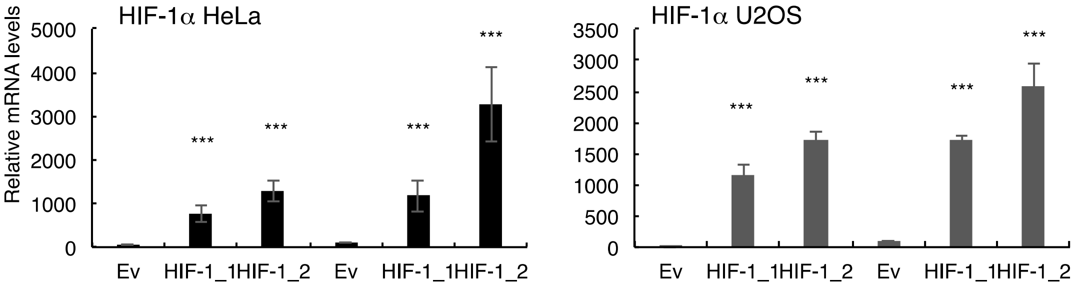

Sup Fig S4 HIF mRNA level controls. A. HeLa and U2OS cells were transfected with the indicated siRNA oligonucleotides for 48 hours prior to RNA extraction. In addition, cells were exposed to 1% O2 for the last 24 hours. Following cDNA synthesis, qPCR analysis was performed for the levels of HIF subunits mRNA. Graphs depict mean and standard deviation from a minimum of three independent experiments performed in duplicate. Student t-test was performed and p values

calculated. * $p < 0.05$, ** $p < 0.01$, *** $p < 0.001$. B. HeLa and U2OS cells were transfected with empty vector (EV), or HIF-1α (1 or 2 μg) for 48 hours prior to RNA extraction. In addition, cells were exposed to 1% O_2 for the last 24 hours. Following cDNA synthesis, qPCR analysis was performed for the levels of HIF-1α mRNA. Graphs depict mean and standard error of mean from a minimum of three independent experiments performed in duplicate. Student t-test was performed and p values calculated. * $p < 0.05$, ** $p < 0.01$, *** $p < 0.001$.

Figure S5

KDM2A and KDM2B promoters and putative HREs

Gene	Species	HRE ACGTG	HRE GCGTG
KDM2A	Human		-143, -186, -1104, -2633
KDM2A	Mouse	-2949	
KDM2A	Rat	-2865	-572, -615, -698, -830, 848, -900, -1250, -1598
KDM2B	Human	-6, -1034, -1287, -2939	
KDM2B	Mouse	-891, -1479	-39
KDM2B	Rat*	-181	
KDM2	Drosophila	-803, -2928	-796, -1198, -2365

Sup Fig S5 HRE annotation in the promoters of KDM2 family in different species. Bioinformatic analysis of the genomes of different species, revealed the presence of several HREs.
